# A Forage Allowance by Forage Type Interaction Impacts the Daily Milk Yield of Early Lactation Dairy Cows

**DOI:** 10.3390/ani13081406

**Published:** 2023-04-19

**Authors:** Adam D. Langworthy, Mark J. Freeman, James L. Hills, David K. McLaren, Richard P. Rawnsley, Keith G. Pembleton

**Affiliations:** 1 Tasmanian Institute of Agriculture, University of Tasmania, Private Bag 3523, Burnie, TAS 7320, Australia; adam.langworthy@utas.edu.au (A.D.L.); mark.freeman57@outlook.com (M.J.F.); james.hills@utas.edu.au (J.L.H.); dmclaren@pinionadvisory.com (D.K.M.); richard.rawnsley@utas.edu.au (R.P.R.); 2 Centre for Sustainable Agricultural Systems and School of Agriculture and Environmental Science, University of Southern Queensland, Toowoomba, QLD 4350, Australia

**Keywords:** forage allocation, grazing intensity, pasture, polyculture, species mixture

## Abstract

**Simple Summary:**

We observed a forage allowance by forage type effect on improvements in the daily milk yield of dairy cows grazing swards sown with perennial ryegrass, white clover and plantain relative to those sown to perennial ryegrass only. Improvements in milk yield were evident at lower forage allowances of 14 to 20 kg of dry matter/cow per day (inclusive), diminishing at the highest allowance of 25 kg of dry matter/cow per day. At the lower forage allowances, energy intake would have been a limiting factor for milk production, potentially highlighting the nutritive advantages of plantain and white clover. Increasing species diversity is a possible strategy for overcoming seasonal nutritive challenges present in perennial ryegrass monocultures.

**Abstract:**

We tested for a forage allowance effect on the milk yield of early lactation dairy cow herds grazing swards sown with perennial ryegrass (*Lolium perenne* L.), white clover (*Trifolium repens* L.) and plantain (*Plantago lanceolata* L.) relative to perennial ryegrass alone. The examined allowances consisted of offering 12, 14, 16, 18, 20 or 25 kg of dry matter (DM)/cow per day of grazeable herbage, with diverse swards sown as mixtures and spatially adjacent monocultures. After adapting cows to their assigned forage type for 8 days, treatment effects on milk yield and composition, blood metabolites (beta-hydroxybutyrate, non-esterified fatty acids and urea concentrations), body weight change, forage intake and selection differentials for forage species and certain nutrients were monitored over 7 days. We confirmed a forage allowance effect on milk yield improvements in dairy cows grazing diverse swards relative to perennial ryegrass monocultures. Improvements in milk yield were evident at forage allowances of 14 to 20 kg of DM/cow per day, diminishing at the highest allowance of 25 kg of DM/cow per day. Improvements in milk yield for the mixture and spatially adjacent monocultures peaked at forage allowances of 18 and 16 kg of DM/cow per day, equalling increases of 1.3 and 1.2 kg of milk/cow per day, respectively.

## 1. Introduction

South-eastern Australian and New Zealand dairy systems are predominantly grazing-based [1], with perennial ryegrass (*Lolium perenne* L.) being the primary sown forage species [2]. A disadvantage of the existing perennial-ryegrass-based feedbase is its strong seasonality of growth and nutritive value [3,4]. Up to 60% of annual forage growth occurs in spring [5], which is associated with the transition from high-nutritive-value vegetative growth to low-nutritive-value reproductive development [6,7]. Spring forage exceeding herd requirements is often conserved as silage or hay, while paddocks retained in the grazing rotation often require pre- or post-grazing mechanical defoliation to achieve correct stubble heights for optimum forage quality and growth [8]. The disadvantages of these strategies include forage conservation costs [9,10] and dry matter (DM) losses [11,12]. An infrequently used strategy to reduce seasonality in nutritive value and to shift growth outside of spring is incorporating alternative perennial forage species into the perennial-ryegrass-based feedbase [13]. The success of this strategy depends on the selection of suitable species for the specific site and situation, rather than simply increasing sward diversity per se [14,15]. Both forb and legume species may have a role in achieving these goals. Milk yield responses to the inclusion of forbs and legumes into perennial-ryegrass-based swards have ranged from being negligible [16,17,18] to increases of 1.3 [19], 1.4 [20], 1.7 [21] and 2.0 kg/cow per day [22]. Differences in forage allowance may explain some of this variability [14], with milk yield improvements restricted to experiments allocating < 25 kg of DM/cow per day of grazeable forage (i.e., forage above a target post-grazing forage biomass residual). Understanding this potential interaction is required to identify the forage allowances at which the inclusion of forbs and legumes into perennial ryegrass-based swards benefit milk production.

A forb with significant potential for many temperate dairying regions is forage-type plantain (*Plantago lanceolata* L.). Not only can plantain achieve equal or greater levels of annual forage production than perennial ryegrass [23,24], but owing to its superior tolerance of hot and dry conditions [25,26], plantain can be especially beneficial in increasing summer–autumn growth [24,27,28]. Relative to perennial ryegrass, plantain herbage typically has a lower structural fibre content [29], which can permit individual cow DM and resultant metabolisable energy (ME) intake to be elevated sufficiently to increase milk production [30]. Plantain herbage is also known to be mineral-rich [31,32,33] and have a negative dietary cation–anion difference [34], which can reduce the incidence of the economically important metabolic disorder hypocalcaemia (milk fever) [35]. Aside from these obvious production advantages, incorporating plantain into swards can have the added benefit of reducing environmental nitrogen (N) losses [22,36].

Improvements in forage nutritive value (i.e., lower fibre and higher digestible protein content) and DM intake can also be achieved by incorporating white clover (*Trifolium repens* L.) into perennial-ryegrass-based swards [37,38]. The realisation of these advantages into increased individual cow milk production necessitate that white clover constitutes more than the typical 10–20% of grazed forage on a DM basis [38,39,40]. Achieving higher white clover levels in swards has the added environmental advantage of increasing biological N fixation [41,42], thus reducing the need for fossil-fuel-based synthetic N fertilisers [43]. A disadvantage of elevating the white clover content in swards is the concomitant increase in bloat risk [44], which must be considered when analysing the cost–benefit of white clover.

It is not yet clearly understood how best to incorporate forbs and legumes into the perennial-ryegrass-based feedbase. Incorporating forbs and legumes in a mixture/polyculture with perennial ryegrass provides each species with the chance to exploit niches within the sward [45,46], maximising their complementary aspects. Alternatively, species may be sown in spatially adjacent monocultures within the same field, which has the potential advantages of minimising interspecies competition and allowing for species-specific fertiliser and herbicide management [20]. A disadvantage of spatially adjacent monocultures is the potentially greater ability of grazing ruminants to exhibit a predilection for specific species, which in the case of legumes could exacerbate the risk of bloat.

Our experiment tested the hypothesis of a forage allowance by forage type interaction effect on improvements in the milk yield of early lactation dairy cows grazing functionally diverse swards sown to perennial ryegrass, white clover and plantain relative to perennial ryegrass only. Both mixtures and spatially adjacent monocultures of these species were evaluated to test the implications of these methods in increasing sward diversity on milk production.

## 2. Materials and Methods

### 2.1. Ethical Statement

All procedures involving cattle were approved by the University of Tasmania Animal Ethics Committee (A0012629).

### 2.2. Experimental Site Description

This experiment was conducted over 15 d during mid spring (September/October 2014) using the More Milk from Forage (MMFF) experimental site [20] at the Tasmanian Institute of Agriculture Dairy Research Facility (41°08′ S, 145°77′ E; 155 m above mean sea level), Elliott, northwest Tasmania, Australia. The location is characterised by a cool–temperate climate and winter-dominant rainfall pattern (mean annual rainfall, 1200 mm). During the 15 d experimental period, mean maximum and minimum daily ambient temperatures were 1.7 °C and 2.0 °C warmer than the long-term 40 year average for this period, while mean daily wind speed was 0.33 m/s lower (Table 1). Mean daily relative humidity and total rainfall deviated minimally from the long-term average. Soil at the experimental site is classified as a clay loam red ferrosol (Humic Etrodox) soil [47,48].

The MMFF field site consisted of ten discrete experimental areas (paddocks), ranging in size between 1.20 and 2.16 ha. Each paddock was divided into three plots of equal area, with each plot occupied by a well-established sward of one of three forage types and all forage types present in each paddock. Forage types had been randomly assigned to plots at sowing (April 2012) and consisted of a perennial ryegrass monoculture (PRG); perennial ryegrass, white clover and plantain mixture (RCPM); and spatially adjacent monocultures (SAM) of perennial ryegrass, white clover and plantain (Supplementary Figure S1). Each SAM plot contained a single strip of each forage species, with white clover always occupying the centre strip. All strips in a SAM plot were of equal width (mean ± SD; 14.3 ± 4.6 m) and ran parallel to each other along the full length of the plot. Cultivars used to construct forage types included perennial ryegrass cv. Base^®^ (AR37, non-toxic endophyte), ladino-type white clover cv. Grassland Kopu II^®^ and forage-type plantain cv. Ceres Tonic^®^. Further details regarding the establishment and management of forage types is provided in Pembleton et al. [20].

### 2.3. Experimental Design and Procedures

The experiment was conducted over 15 d and consisted of 72 early lactation dairy cows allocated equally between 18 treatments, with treatments including each combination of the three forage types (described above) by six forage allowance levels. Each treatment was allocated four early lactation dairy cows for the experiment duration, with previous research showing this lactation stage to be when the effects of forage type on milk production are greatest [20]. A treatment herd size of four was selected to give a high probability of detecting treatment effects (power = 0.8). Power analysis was conducted as outlined by Cohen [49] assuming a large treatment effect via the pwr.f2.test function in R (*df*_1_ = 17, *df*_2_ = 54, Cohen’s *f*^2^ = 0.35, *p* = 0.05) [50].

**Figure 1:**
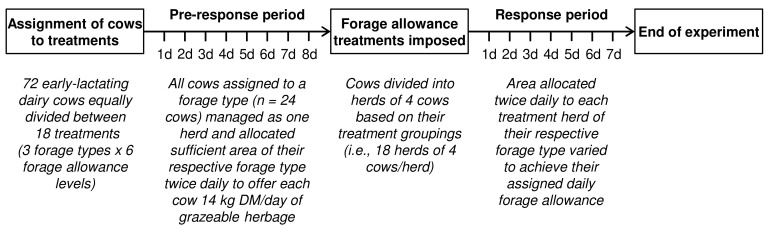
Schematic diagram of experimental sequence.

Forage allowance treatments were imposed for the final 7 d of the experiment (response period), with cows offered 12, 14, 16, 18, 20 or 25 kg of DM/cow per day of grazeable herbage from their respective forage type. An 8 d pre-response period preceded the response period, when all cows were allocated 14 kg of DM/cow per day of their respective forage type to facilitate adaptation of the rumen. During the pre-response period, all cows assigned to a forage type were managed as one herd (i.e., three herds of 24 cows), whereas during the response period (when forage allowance treatments were imposed), cows were managed in their treatment herds (i.e., 18 herds of 4 cows) (Figure 1). After each daily milking event (0700 and 1500 h), cows received half of their daily forage allowance. During each of these grazing periods, all cows grazed within the same paddock, with portable electric fencing (Kiwitech Ltd., Bulls, New Zealand) used during the response period to divide forage type plots crosswise into forage allowance subplots. This resulted in the treatment herds grazing SAM subplots being offered equal areas of each species component. The ordering of forage allowance subplots in each plot was randomly determined, with forage allowances achieved by manipulating the size of the subplots provided to treatment herds between 72.5 and 374.4 m^2^.

During the week preceding the experiment, each plot (*n* = 30) was yield-mapped at 2 m intervals using a C-Dax pasture meter integrated with a GPS console (C-Dax Ltd., Palmerston North, New Zealand). Manifold GIS software was then used to construct a yield map [51]. A day before cows grazed a new paddock, eight randomly placed 0.5 by 0.5 m quadrats were cut to ground level in the PRG plot, RCPM plot and each species component of the SAM plot. Cut material was removed and immediately weighed, with DM content (%) determined (for experiment management purposes only) by repeatedly drying a subsample of known weight in a microwave oven until constant weight was achieved. Pre-grazing forage biomass (kg of DM/ha) of each quadrat was then calculated and integrated into the yield map to determine the required subplot area for each treatment cow herd. Forage was allocated above a post-grazing residual of 1250 kg of DM/ha for the PRG, RCPM and perennial ryegrass monoculture component of the SAM; 500 kg of DM/ha for the white clover monoculture component of the SAM; and 800 kg of DM/ha for the plantain monoculture component of the SAM.

### 2.4. Animals

Each treatment herd consisted of four early lactation multiparous crossbred dairy cows (*Bos taurus*). Cows in each treatment herd were balanced for age (mean ± SD; 4.7 ± 0.5 years old), milk production (mean ± SD; 32.3 ± 3.9 kg/cow per day), days in milk (mean ± SD; 38.6 ± 3.7 d), body weight (mean ± SD; 497 ± 55 kg) and breed. An in-bail feeding system supplied each cow with 4 kg of DM/d of concentrate (wheat-based pellet with additional mineral and vitamin supplements). The daily concentrate allowance was equally split between morning and afternoon milking events, and consisted of the following: crude protein (CP), 11.2% of DM; neutral detergent fibre (NDF), 12.8% of DM; acid detergent fibre (ADF), 6.0% of DM; lignin, 1.8% of DM; water-soluble carbohydrates (WSC), 1.8% of DM; crude fat, 2.1% of DM; and estimate metabolisable energy (ME), 12.5 MJ/kg of DM. During grazing, each cow had ad libitum access to bloat blocks that contained 10% alcohol ethoxylate teric 12A 23 (Bloat-Liq, Olsson’s Industries, Melbourne, VIC, Australia). Furthermore, white clover monoculture components of the SAM treatment were sprayed immediately before grazing with 15 L/ha of an emulsifiable anti-bloat oil (BP Pasture spray, BP Australia Pty Ltd., Melbourne, Australia). All cows had ad libitum access to fresh water while grazing via Kiwitech portable 100 L water troughs (WT R100).

### 2.5. Measurements

At each milking event, milk yield (kg) of each cow was recorded by a DeLaval Alpro milk metering system (DeLaval International AB, Tumba, Sweden). On the day preceding (covariate) and the final three days of the response period, milk samples were collected at both daily milking events. Milk samples were analysed by TasHerd Pty Ltd. (Haspen, Tasmania, Australia) using a Bentley B2000 Infrared Milk Analyzer (Bentley Instruments Inc., Chaska, MN, USA) for fat, true protein and lactose concentration. At each milking event (i.e., twice daily), automatic walk-over scales (DeLaval AWS100 automatic weighing system) recorded body weight as cows exited the milking parlour. Twice-daily weighing of cows permitted for the effect of changes in rumen fill on daily body weight measurements to be averaged. Blood samples were collected from each cow via coccygeal venepuncture using 10 mL Vacutainers^®^ (Becton, Dickinson and Company, Plymouth, UK) containing sodium heparin. Samples were collected after morning milking events on the day preceding (covariate) and for each of the final four response period days. Blood samples were centrifuged (1125× *g*) for 10 min at 4 °C, with plasma then collected and frozen at −20 °C until laboratory analysis. Plasma was analysed by the Western Australian Department of Agriculture and Food Animal Health Laboratory (South Perth, Western Australia, Australia) using an Olympus AU400 Chemistry Analyser (Olympus, Tokyo, Japan) for blood urea nitrogen (BUN), beta-hydroxybutyrate (BHBA) and non-esterified fatty acid (NEFA) concentrations.

Immediately pre- and post-grazing, forage biomass in each subplot was estimated from compressed forage height (mm), measured with an electronic rising plate meter (Farmworks Systems, Fielding, New Zealand). Compressed forage height values were converted to forage biomass on a hectare basis (kg of DM/ha) using separate linear regression equations for PRG and RCPM treatments, and for each species component of the SAM treatment [52]. Each calibration equation was developed in the week preceding the experiment by measuring the compressed height of forage contained in 80 randomly selected square quadrant (0.25 m^2^) samples. Forage in each quadrant was harvested to ground level and immediately weighed, with a subsample of known weight dried to constant weight at 60 °C in a fan-forced drying oven (Unitherm drying oven; S & T Engineering Company, Birmingham, UK). Dried subsamples were reweighed to determine DM content and calculate forage biomass (kg of DM/ha). Regression equations were then developed, with forage biomass as the dependent variable and compressed forage height as the independent variable (Supplementary Table S1).

Forage intake from each subplot was estimated from the pre- and post-grazing estimates of forage biomass using the following equation:

Estimated forage intake (kg of DM/cow per day) = (M_pre_ − M_post_)/4,

where M_pre_ = pre-grazing forage biomass (kg of DM) and M_post_ = post-grazing forage biomass (kg of DM). Daily estimated forage intake was the sum of estimated forage intake from morning and evening grazing periods (i.e., the two subplots allocated daily to the treatment herd). Predicted forage intakes were calculated empirically using forage nutritive value data and known energy requirements for maintenance, body weight change, pregnancy and production [53].

Prior to grazing a paddock, hand-cut forage samples were collected from each forage type plot to determine the botanical composition and nutritive value of offered forage. In both PRG and RCPM plots, all forage above ground level was collected from twenty-five points (40 by 40 mm) along a defined transect. This sampling process was repeated for each species component of SAM plots. On the day preceding (covariate) and the final four days of the response period, forage samples were similarly collected from each subplot immediately pre- and post-grazing. Samples were used in conjunction with forage biomass data to calculate selection differentials exhibited by treatment herds for the sown forage species and certain nutritive value parameters, including CP, NDF and ADF. Botanical composition was determined by hand-separating forage samples into their individual botanical components (perennial ryegrass, white clover, plantain and other volunteer species (weeds)), which were dried at 60 °C for 72 h. Each dried botanical component was weighed to calculate botanical composition on a DM basis. Forage for nutritive value analysis was similarly dried and then milled through a 1 mm screen before being assayed. All nutritive value samples were analysed via wet chemistry procedures by the Dairy One Forage Laboratory (Ithaca, New York, NY, USA). In vitro DM digestibility (IVDMD) was determined using an ANKOM Daisy*^II^* Incubator (ANKOM Technology Corporation, Fairport, NY, USA), with dry-milled forage samples anaerobically incubated at 39 °C for 48 h in a medium containing Van Soest buffer solution and rumen fluid [54,55]. Rumen fluid was collected from fistulated lactating dairy cows, which were total mixed ration (TMR)-fed. Further details regarding these procedures and an estimation of ME are provided in Pembleton et al. [20]. Selection differentials for selected forage species and nutritive parameters were calculated as the ratio of the species or nutrient concentration in herbage consumed relative to the herbage on offer using the following equation, as described by Jacobs et al. [56]:

Selection differential = N_sel_/N_pre_

where N_sel_ = [(M_pre_ × N_pre_) − (M_post_ × N_post_)]/(M_pre_ − M_post_), where M_pre_ = pre-grazing forage biomass (kg of DM), M_post_ = post-grazing forage biomass (kg of DM), N_pre_ = pre-grazing species component or nutrient concentration (g/kg DM) and N_post_ = post-grazing species component or nutrient concentration (g/kg DM).

### 2.6. Statistical Analysis

Effects of forage allowance and forage type on milk yield and composition, body weight change, blood metabolite levels and estimated forage intake were examined using multiple regressions analysis. Separate regression models were developed for each measured parameter, with the choice of regression manually pre-set based on graphical observations of the data. Forage allowance was included in all models as a continuous predictor (explanatory) variable, with individual cows being the unit of analysis. Forage type was only included in models as a categorical predictor variable if it statistically significantly improved the variance accounted for by the model. This was determined by an ANOVA comparison of models developed for each measured parameter with and without the inclusion of forage type. Inclusion of forage type in the model resulted in the generation of a separate regression equation for each forage type. Despite using historical milk production records to balance treatment herds, significant treatment herd differences in milk yield and composition were detected in data collected immediately before commencement of the experiment. Consequently, these values were used as covariates prior to fitting multiple regressions to these measurements.

Pre- and post-grazing forage biomass and selection differentials were analysed via split-plot ANOVA, with forage type the main plot, forage allowance the subplot and the monitored response days treated as blocks. The split-plot ANOVA statistical model was as follows:

$$Y_{ijk}=\mu +\alpha_{i}+\gamma_{k}+\eta_{ik}+\beta_{j}+{\left(\alpha \beta \right)}_{ij}+\varepsilon_{ijk}$$

in which $Y_{ijk}$ is the measured parameter (forage biomass or selection differential), $\alpha_{i}$ is the fixed effect of forage type, $\gamma_{k}$ is the fixed effect of response day (block), $\eta_{ik}$ is the whole-plot error, $\beta_{j}$ is the fixed effect of forage allowance, ${\left(\alpha \beta \right)}_{ij}$ is the interaction between forage type and forage allowance and $\varepsilon_{ijk}$ is the split-plot error.

All statistical analyses were undertaken using the R statistical package (R Core Team 2014). Unless otherwise stated, differences discussed are significant at *p* < 0.05.

## 3. Results

### 3.1. Botanical Composition and Nutritive Value

Forage offered to cows grazing PRG and the perennial ryegrass component of the SAM treatment was greater than 97% perennial ryegrass, with the remainder being weeds (Table 2). Forage offered in the RCPM treatment was approximately two-thirds perennial ryegrass, one-third plantain, with white clover and weeds being only minor components (1.6 and 4.1%, respectively). While the forage offered in the plantain component of the SAM treatment was primarily plantain (77.8%), there was a sizable weed component (17.4%). Weeds contributed 52.3% of the offered forage in the white clover component of the SAM treatment.

Analysis of the nutritive content of offered forage (Table 2) revealed that herbage obtained from the white clover component of the SAM treatment contained the lowest fibre (NDF and ADF) concentrations and the highest available protein concentration, IVDMD and estimated ME content. The forage offered in PRG and the perennial ryegrass component of the SAM treatment had the highest NDF concentration. Compared to these perennial ryegrass monocultures, the forage offered in the RCPM treatment had lower NDF and higher lignin and WSC contents. This reflected the inclusion of plantain in the RCPM treatment, as evidenced by the nutritive value of the plantain component of the SAM treatment. High WSC levels resulted in the forage offered in the RCPM and plantain component of the SAM treatment having the highest WSC:CP ratios (≥0.61). White clover contributed minimally to the nutritive value of the RCPM treatment, as it only represented 1.6% of the offered forage. Of all forages, the plantain component of the SAM treatment had the lowest estimated ME content, reflecting its higher ash and lignin content and resultantly lower IVDMD than all other forages. Supplementary Table S2 contains additional details on the chemical composition of each forage type.

### 3.2. Milk Yield and Composition

**Figure 2:**
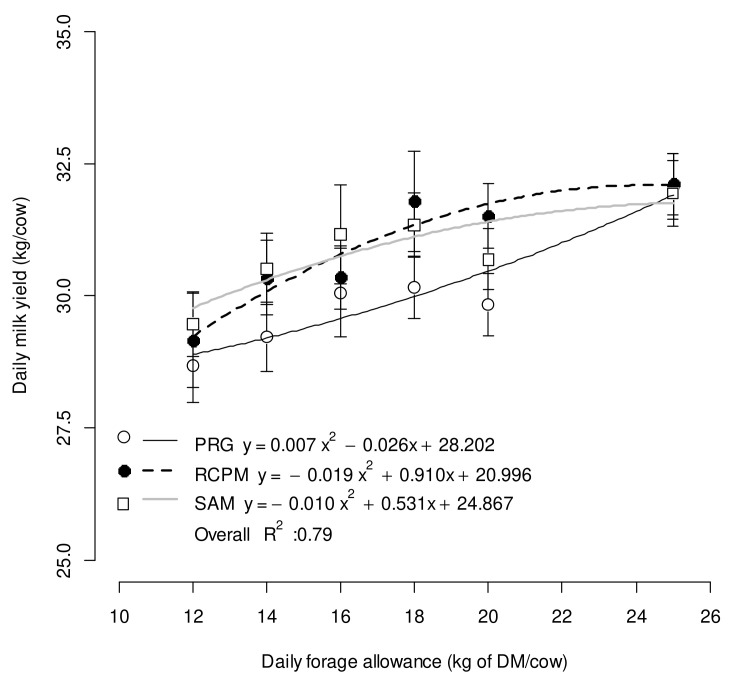
Milk yield (kg/cow per day) response of early lactation dairy cows to increasing allowances (kg of DM/cow per day) of different forage types, including PRG, perennial ryegrass monoculture (solid black line); RCPM, perennial ryegrass, white clover and plantain mixture (broken black line); and SAM, spatially adjacent monocultures of perennial ryegrass, white clover and plantain (grey line). As forage type significantly improved the variance accounted for by the model, separate forage type relationships are shown. Symbols are means ± SEM (*n* = 4).

Across all treatments, the milk harvested during the experiment consistently contained 4.8% fat, 3.0% protein and 5.1% lactose. Milk yield increased as forage allowance increased (Figure 2). At *p* = 0.08 significance level, we observed a forage type effect on this relationship. Fitted regressions predicted that the milk yield of cows grazing RCPM and SAM treatments would peak at forage allowances of 24.5 and 25 kg of DM/cow per day, respectively. At the highest tested forage allowance of 25 kg of DM/cow per day, the milk yield of cows grazing the PRG treatment was still increasing. At forage allowances between 14 and 20 kg of DM/cow per day (inclusive), cows produced up to 1.3 and 1.2 kg/cow per day more milk when grazing RCPM or SAM treatments relative to the PRG treatment, respectively. The greatest difference in milk yield between RCPM and SAM vs. PRG treatments occurred at forage allowances of 18 and 16 kg of DM/cow per day, respectively.

### 3.3. Blood Metabolite Levels

**Figure 3:**
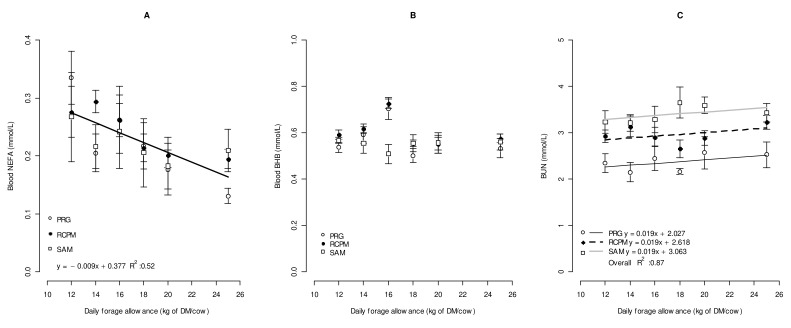
(**A**) Non-esterified fatty acid (NEFA); (**B**) beta-hydroxybutyrate (BHB); and (**C**) blood urea nitrogen (BUN) concentrations in the blood of early lactation dairy cows grazing three different forage types at six forage allowances. Forage types included PRG, perennial ryegrass monoculture; RCPM, perennial ryegrass, white clover and plantain mixture; and SAM, spatially adjacent monocultures of perennial ryegrass, white clover and plantain. Separate forage type relationships (**C**) are only shown when the addition of this factor improved the variance accounted for by the model. No regression could be fitted to BHB data (**B**). Symbols are means ± SEM (*n* = 4).

Blood NEFA concentration decreased as forage allowance increased (Figure 3A), with this relationship being independent of forage type. In contrast, blood BHB concentrations were independent of both forage type and allowance (Figure 3B), averaging 0.57 mmol/L. The concentration of BUN increased as forage allowance increased (Figure 3C), with the rate of increase consistent across all forage types. At each forage allowance, BUN concentrations for cows grazing the RCPM and SAM treatments were 0.591 and 1.036 mmol/L higher than their contemporaries grazing the PRG treatment, respectively. 

### 3.4. Body Weight Change

The body weights of cows grazing RCPM and SAM treatments did not decline (Table 3). At forage allowances ≤ 18 kg of DM/cow per day, the body weight of cows grazing the PRG treatment declined by between 0.4 and 0.7 kg/cow per day.

### 3.5. Forage Intake and Selection Differentials

Within each forage type (including each species component of the SAM treatment), pre-grazing forage biomass was similar across tested forage allowances (Table 4). As forage allowance increased, cows left greater levels of post-grazing forage biomass. As would be expected from the target residuals, post-grazing forage biomass was lower in the white clover relative to the plantain component of the SAM treatment, with both components having a lower post-grazing forage biomass than the PRG, RCPM or perennial ryegrass components of the SAM treatment.

**Figure 4:**
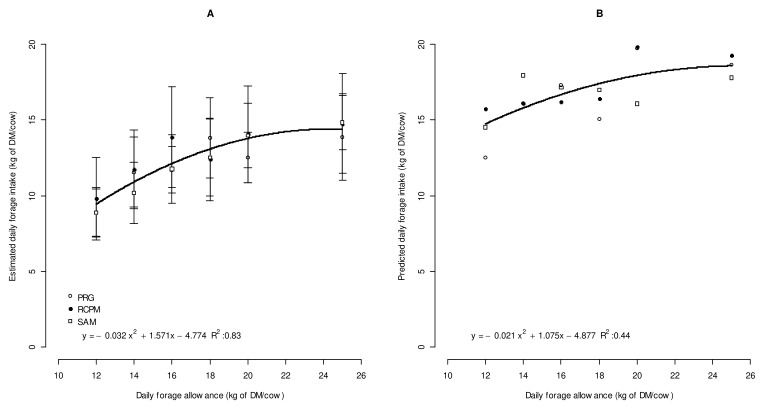
(**A**) Impact of forage allowance on estimated forage intake (estimated from pre- and post-grazing forage biomass) and (**B**) predicted forage intake (predictions based on the equations in CSIRO [53]) of three forage types, including PRG, perennial ryegrass; RCPM, perennial ryegrass, white clover and plantain mixture; and SAM, spatially adjacent monocultures of perennial ryegrass, white clover and plantain. Symbols are means ± SEM (*n* = 4) for estimated forage intake, while symbols for predicted forage intake represent overall values for the experimental period. Solid lines show the overall regression models developed between explanatory and outcome variables.

The estimated forage intake was independent of forage type and increased at greater forage allowances (Figure 4A), although the rate of increase declined as forage allowance increased. The fitted regression predicted that the maximum estimated forage intake (14.4 kg of DM/cow per day) occurred at a forage allowance of 24.5 kg of DM/cow per day. Estimated forage intake averaged 4.5 kg of DM/cow per day less than the predicted forage intake (Figure 4A,B).

Across each forage type, cows receiving forage allowances of 18, 20 or 25 kg of DM/cow per day exhibited greater selectivity for perennial ryegrass than cows allocated 16 kg of DM/cow per day (Table 5). In contrast, cows grazing RCPM and SAM forage types exhibited greater selectivity for plantain at lower forage allowances. Within RCPM and SAM treatments, cows consistently showed a selective preference for white clover (mean selection differential, 1.57).

Calculated selection differentials for CP were independent of forage type or allowance (Table 6). Cows showed minimal selective preference for CP, as indicated by the mean selection differential across treatments being close to 1 (1.06). Cows grazing the SAM, relative to PRG treatment, exhibited a significantly greater selection against NDF. At forage allowances of 20 and 25 kg of DM/cow per day, cows grazing the RCPM and SAM treatments showed a greater selection against ADF than cows grazing the PRG treatment. At a forage allowance of 12 kg of DM/cow per day, cows again showed a greater selection against ADF when grazing the SAM relative to the PRG treatment.

## 4. Discussion

This experiment confirmed our hypothesis of a forage allowance by forage type interaction effect on improvements in the daily milk yield of dairy cows grazing functionally diverse swards (RCPM and SAM) relative to perennial ryegrass monocultures (PRG). Such effects were independent of milk composition, which across the evaluated forage allowance by forage type treatments consistently contained 4.8% fat, 3.0% protein and 5.1% lactose. The experiment was conducted in spring, which is known from previous experiments to be the seasonal period when feeding evaluated diverse swards to spring-calving dairy cows has the greatest chance of increasing their daily milk production above that possible with perennial ryegrass only [20]. In the current experiment, such milk yield advantages of the diverse forage types were only evident at forage allowances resulting in below maximum estimated forage intake (i.e., forage allowances < 24.5 kg of DM/cow per day). Maximum daily milk yield improvements occurred at forage allowances of 18 kg of DM/cow per day for the RCPM and 16 kg of DM/cow per day for the SAM, equalling daily milk yield increases of 1.3 and 1.2 kg/cow per day, respectively. The advantages of the diverse forage types for body weight change followed a similar pattern to daily milk yield, with forage allowances ≤ 18 kg of DM/cow per day resulting in the cows grazing the PRG treatment losing weight, whereas cows grazing the RCPM and SAM treatments gained weight.

The energy intake of cows with an estimated forage intake below maximum would have been a limiting factor for milk production, potentially highlighting the nutritive value advantages of the diverse forage types. Assuming that cows receiving the highest forage allowance of 25 kg of DM/cow per day ate to requirements, cows allocated between 12 and 20 kg of DM/cow per day had an average forage intake deficit of 2.4 kg of DM/cow per day. The results of this calculation are supported by blood NEFA concentrations, which were higher for cows allocated less forage, indicating a more significant lipolysis of adipose tissue to supply energy [57].

Our study was not a highly controlled confinement-based feeding study (e.g., individual metabolic stalls), but instead compared the milk production potential of evaluated forage types when grazed to provided commercially relevant results for grazing-based dairy systems. A consequence of grazing systems is that cows may not always eat their full allocation and may exhibit a predilection for certain forage species present in the sward. At the lower forage allowances associated with the diverse forage types having a milk production benefit, cows grazing the RCPM and SAM treatments consumed relatively more plantain (selection differential data). While this could have simply resulted from cows grazing further into swards, where relatively more plantain may have been present, grazing at lower forage allowances would have resulted in plantain having a greater potential to influence cow production.

A benefit of including plantain in the RCPM treatment was an increase in the WSC:CP ratio of forage above PRG levels (0.61 vs. 0.50), as plantain contributed 26.5% of offered forage DM in this treatment. Evidence is provided by the higher WSC:CP ratio of forage obtained from the plantain component of the SAM relative to PRG treatment (0.69 vs. 0.50). This may have improved the efficiency of N conversion into microbial protein, due to the improved synchrony of energy and protein within the rumen [58,59]. Indeed, the WSC:CP ratio for the plantain component of the SAM treatment was only slightly below the benchmark (>0.70) known to significantly improve ruminant N utilization efficiency [59,60]. Advantages of white clover were not evident in the RCPM treatment because of the minimal contribution of white clover on a DM basis (1.6%). Lower temperatures early in the growing season, as is typical for the experimental site, would explain the low prevalence of clover during our experiment [61]. This would also help to explain the high weed burden and lower-than-desired prevalence of clover in the white clover component of the SAM treatment (47.7% on a DM basis). The nutritive advantages of the white clover component of the SAM relative to PRG treatments thus cannot be attributed solely to white clover but included a higher estimated ME (11.5 vs. 11.3 MJ/kg of DM) and available protein content (23.1 vs. 15.8%).

Across forage types, selection differential values for CP were similar and close to one, indicating that cows did not exhibit any selection for or against CP. An explanation is provided by BUN concentrations remaining within the expected range of 2–4 mmol/L for morning-sampled dairy cows in grazing systems with adequate CP intake [62]. Recorded BUN levels were lower than levels often prescribed in the literature for cows receiving an adequate CP supply because cows used in these studies were TMR-fed [63,64]. Unlike TMR-fed cows with regular access to feed, cows in grazing systems experience a period of fasting overnight due to forage depletion, which is well known to depress BUN concentrations [65,66].

The inclusion of plantain and/or white clover in the diverse forage types resulted in offered forage having a lower fibre (NDF and ADF) content than the PRG treatment and permitted cows to select a diet lower in fibre. Reduced fibre intake is typically associated with an increased digestibility of consumed forage, decreased rumen retention time and a concomitant increase in voluntary forage intake [67,68]. Forage obtained from the white clover component of the SAM had a greater IVDMD than the PRG treatment, but the opposite was true for the plantain component. Forage in the RCPM relative to PRG treatment subsequently had a lower IVDMD due to containing a significant plantain component. These observations can be partly explained by the higher lignin and ash contents of plantain relative to perennial ryegrass forage [69]. However, higher lignin content in plantain forage may have been advantageous in maintaining the reservoir of buffering exchangeable cations in the rumen and the provision of coarse fibre necessary for rumen function [70]. The lower IVDMD of plantain forage may also stem from the rumen fluid used for this assessment being obtained from TMR-fed donor cows. Previous research showed that rumen fluid obtained from TMR vs. pasture-fed donor cows resulted in 19.4% lower IVDMD values for pasture samples [71]. The use of rumen fluid from unacclimated cows may have been particularly problematic for assessing the IVDMD of plantain herbage, as plantain forage contains antimicrobial compounds known to interfere with ruminal fermentation [26].

Despite the white clover component of the SAM treatment having the highest IVDMD of all forages, the estimated forage intake remained independent of forage type. This observation contrasts previous research showing that legumes are often associated with higher forage intake than grasses [72]. This discrepancy may result from the considerable intratreatment variation for estimated forage intake (as indicated by the error bars on Figure 4A). Differences in predicted forage intake amongst the forage types and allowances (Figure 4B) ranged between 3.6 and 0.1 kg of DM/cow per day. Low differences would be challenging to detect using pre- and post-grazing biomass measures with a rising plate metre. Unfortunately, animal-based methods (e.g., N-alkanes or other tracers), while potentially more accurate, are generally unsuitable for use in diverse swards [73].

Across forage types there was a diminishing rate of increase in estimated forage intake with increases in forage allowance. Similar responses were reported by Moate et al. [74] and Peyraud et al. [75], with our observations in line with the theoretical response of forage intake to forage allowance proposed by Minson [76]. Our observation of maximum estimated forage intake occurring at a forage allowance of 24.5 kg of DM/cow per day is on the lower end of the range reported in previous studies [74,75,76]. Many factors other than forage allowance influence forage intake, including supplementary feeding level, cow size and prevailing environmental conditions [77], preventing direct comparison between experiments. Sward structure and species composition are also known to affect forage intake by influencing both bite size and rate [77].

## 5. Conclusions

This experiment has confirmed our hypothesis of a forage allowance effect on improvements in the daily milk yield of cows grazing functionally diverse swards relative to perennial ryegrass monocultures. Advantages were evident at lower forage allowances, when energy intake was limiting for milk production, potentially highlighting the nutritive value advantages of these diverse forage types. The most significant milk production benefits occurred at forage allowances between 16 and 18 kg of DM/cow per day. These forage allowance levels align with current grazing practices and consequently the successful integration of either RCPM or SAM treatments into grazing-based dairy systems will not require changes in daily forage allowance.

## Figures and Tables

**Table 1:** Environmental conditions experienced during the 15 d experimental period along with long-term 40-year means for this period. Long-term data sourced from the SILO climate database: http://www.longpaddock.qld.gov.au/silo/ (accessed 17 June 2015).

Parameter	Experiment	Long-Term Mean
Mean daily ambient temperature (°C)	11.2	9.4
Mean daily maximum ambient temperature (°C)	14.9	13.2
Mean daily minimum ambient temperature (°C)	7.4	5.4
Mean daily relative humidity (%)	81.5	80.7
Mean daily maximum relative humidity (%)	94.6	96.9
Mean daily minimum relative humidity (%)	68.5	64.6
Mean daily wind speed (m/s)	1.65	1.98
Number of rain days (d)	10	9
Total rain received (mm)	52.2	55.7

**Table 2:** Botanical composition (% species on a DM basis) and nutritive value of forage offered to cows grazing each forage type, including PRG, perennial ryegrass monoculture; RCPM, perennial ryegrass, white clover and plantain mixture; and SAM, spatially adjacent monocultures of perennial ryegrass, white clover and plantain. Values presented are means ± SE (*n* = 10). Abbreviations include ADICP, acid detergent insoluble crude protein and ME, metabolisable energy.

Item	PRG	RCPM	SAM		
			Perennial Ryegrass	White Clover	Plantain
Species					
Perennial ryegrass (%)	98.7 ± 0.9	67.9 ± 6.1	97.7 ± 1.5	0.0 ± 0.0	4.0 ± 1.4
White clover (%)	0.0 ± 0.0	1.6 ± 2.2	0.0 ± 0.0	47.7 ± 19.2	0.8 ± 2.4
Plantain (%)	0.0 ± 0.0	26.5 ± 5.5	0.0 ± 0.0	0.0 ± 0.0	77.8 ± 4.1
Weeds (%)	1.3 ± 0.9	4.1 ± 3.0	2.3 ± 1.5	52.3 ± 6.1	17.4 ± 3.4
DM content (%)	17.3 ± 0.7	14.7 ± 0.5	16.9 ± 0.6	13.2 ± 0.7	12.2 ± 0.4
Nutritive value					
Crude protein (%)	16.5 ± 0.5	18.2 ± 0.4	16.9 ± 0.5	25.3 ± 0.5	18.4 ± 0.6
Available protein (%)	15.8 ± 0.5	16.4 ± 0.5	16.1 ± 0.5	23.1 ± 0.4	15.3 ± 0.5
ADICP (%)	0.8 ± 0.2	1.7 ± 0.3	0.8 ± 0.2	2.2 ± 0.3	3.2 ± 0.4
Soluble protein (% of CP)	31 ± 0.8	29.1 ± 0.7	31 ± 0.7	32.2 ± 0.8	25.5 ± 0.7
Acid detergent fibre (%)	27.2 ± 0.5	27.2 ± 0.5	27.3 ± 0.5	23.9 ± 0.6	26.1 ± 0.6
Neutral detergent fibre (%)	45.6 ± 0.5	40.6 ± 0.5	45.7 ± 0.4	31.6 ± 0.8	33.8 ± 0.8
Lignin (%)	2.5 ± 0.3	4.3 ± 0.4	2.6 ± 0.3	5.0 ± 0.6	7.2 ± 0.4
Water soluble carbohydrates (%)	9.0 ± 0.4	11.1 ± 0.5	8.9 ± 0.4	9.0 ± 0.4	12.7 ± 0.4
Crude fat (%)	3.2 ± 0.2	3.1 ± 0.2	3.1 ± 0.4	2.8 ± 0.2	2.8 ± 0.2
Ash (%)	9.9 ± 0.3	10.7 ± 0.3	10.3 ± 0.3	10.7 ± 0.3	11.8 ± 0.3
In vitro DM digestibility (%)	76.3 ± 0.5	75.6 ± 0.6	74.0 ± 0.8	77.4 ± 0.6	72.9 ± 0.6
Estimated ME (MJ/kg of DM)	11.3 ± 0.2	11.2 ± 0.2	10.9 ± 0.3	11.5 ± 0.3	10.7 ± 0.2

**Table 3:** Daily body weight change of early lactation dairy cows grazing three different forage types at six forage allowances. Forage types included PRG, perennial ryegrass monoculture; RCPM, perennial ryegrass, white clover and plantain mixture; and SAM, spatially adjacent monocultures of perennial ryegrass, white clover and plantain. As forage type significantly improved the variance accounted for by the model, separate forage type relationships are shown for each forage type.

	Forage Allowance (kg of DM/cow per Day)						Regression	Overall R^2^
	12	14	16	18	20	25		
Forage type	Daily body weight change (kg/cow per day)							
PRG	−0.4	−0.6	−0.7	−0.5	0.2	0.1	y = 0.06x − 1.33	0.76
RCPM	0.7	0.1	0.6	0.1	0.5	0.5	y = 0.06x − 0.62	
SAM	1.1	1.5	2.5	1.2	2.1	2.4	y = 0.06x + 0.63	

**Table 4:** Pre- and post-grazing forage biomass (kg of DM/ha) of three forage types allocated to early lactation dairy cows at six forage allowances. Forage types included PRG, perennial ryegrass monoculture; RCPM, perennial ryegrass, white clover and plantain mixture; and SAM, spatially adjacent monocultures of perennial ryegrass, white clover and plantain. Values with different scripts are significantly different (*p* < 0.05).

	Forage Allowance (kg of DM/Cow per Day)						
	12	14	16	18	20	25	Mean
**Pre-grazing forage biomass**							
Forage type							
PRG	2867	2953	2940	3058	3077	3028	2987 b
RCPM	3156	3073	3174	3030	3046	3023	3084 b
SAM Ryegrass	2847	2806	2812	2838	2991	2932	2871 b
White clover	2040	2111	2211	2113	2260	2233	2161 a
Plantain	2838	2867	2894	2872	3081	2955	2918 b
Mean	2750	2762	2806	2782	2891	2834	
Effects:	Forage type:		*p* < 0.01	SED:			206
	Forage allowance:		*p* > 0.05	SED:			46
	Interaction:		*p* > 0.05	SED (Within forage type):			104
				SED (Between forage types):			227
**Post-grazing forage biomass**							
Forage type							
PRG	1590	1593	1659	1765	1950	1999	1759 c
RCPM	1731	1592	1658	1776	1799	1966	1754 c
SAM Ryegrass	1505	1571	1647	1632	1618	1786	1627 c
White clover	1178	1133	1080	1214	1328	1392	1221 a
Plantain	1245	1273	1273	1397	1579	1639	1401 b
Mean	1450 a	1432 a	1463 a	1557 b	1655 c	1756 c	
Effects:	Forage type:		*p* < 0.001	SED:			73
	Forage allowance:		*p* < 0.001	SED:			37
	Interaction:		*p* > 0.05	SED (Within forage type):			84
				SED (Between forage types):			105

**Table 5:** Selection differentials for perennial ryegrass, white clover and plantain by early lactation dairy cows grazing three forage types at six forage allowances. Forage types included PRG, perennial ryegrass monoculture; RCPM, perennial ryegrass, white clover and plantain mixture; and SAM, spatially adjacent monocultures of perennial ryegrass, white clover and plantain. Values with different scripts are significantly different (*p* < 0.05). At the interaction level, values with different upper-case scripts differ within a forage allowance, while values with different lower-case scripts differ within a forage type.

Forage Allowance (kg of DM/Cow per Day)	12	14	16	18	20	25	Mean
**Perennial Ryegrass**							
Forage type							
PRG	1.03	1.00	0.87	0.99	0.95	0.99	0.97
RCPM	0.79	0.72	0.70	1.13	1.15	1.57	1.01
SAM	1.31	1.10	0.82	1.20	1.36	1.26	1.17
Average	1.04 abc	0.94 ab	0.79 a	1.11 bc	1.15 bc	1.27 c	
Effects	Forage type:		*p* > 0.05	SED:			0.086
	Forage allowance:		*p* < 0.05	SED:			0.141
	Interaction:		*p* > 0.05	SED (Within forage treatment):			0.245
				SED (Between forage treatment):			0.239
**White Clover**							
Forage type							
PRG	NA	NA	NA	NA	NA	NA	NA
RCPM	1.57	0.99	1.60	0.78	1.55	1.37	1.31
SAM	1.94	1.64	2.03	1.72	2.04	1.65	1.84
Average	1.77	1.31	1.81	1.25	1.80	1.51	
Effects:	Forage type:		*p* > 0.05	SED:			0.232
	Forage allowance:		*p* > 0.05	SED:			0.253
	Interaction:		*p* > 0.05	SED (Within forage treatment):			0.438
				SED (Between forage treatment):			0.462
**Plantain**							
Forage type							
PRG	NA	NA	NA	NA	NA	NA	NA
RCPM	2.13	1.93	2.01	1.58	1.27	1.14	1.68
SAM	1.65	2.00	2.06	1.32	1.12	1.07	1.54
Average	1.89 b	1.97 b	2.03 b	1.45 ab	1.20 a	1.10 a	
Effects:	Forage type:		*p* > 0.05	SED:			0.394
	Forage allowance:		*p* < 0.05	SED:			0.316
	Interaction:		*p* > 0.05	SED (Within forage treatment):			0.447
				SED (Between forage treatment):			0.567

**Table 6:** Selection differentials for crude protein, neutral detergent fibre and acid detergent fibre by early lactation dairy cows grazing three forage types at six forage allowances. Forage types included PRG, perennial ryegrass monoculture; RCPM, perennial ryegrass, white clover and plantain mixture; and SAM, spatially adjacent monocultures of perennial ryegrass, white clover and plantain. Values with different scripts are significantly different (*p* < 0.05). At the interaction level, values with different upper-case scripts differ within a forage allowance, while values with different lower-case scripts differ within a forage type.

Forage Allowance (kg of DM/Cow per Day)	12	14	16	18	20	25	Mean
**Crude protein**							
Forage type							
PRG	1.14	1.29	1.07	0.99	0.94	1.10	1.09
RCPM	1.08	0.97	1.01	1.06	1.22	1.10	1.07
SAM	1.04	1.04	0.99	1.01	0.97	1.01	1.01
Mean	1.09	1.10	1.02	1.02	1.04	1.07	
Effects:	Forage type:		*p* > 0.05	SED:			0.098
	Forage allowance:		*p* > 0.05	SED:			0.057
	Interaction:		*p* > 0.05	SED (Within forage type):			0.099
				SED (Between forage types):			0.133
**Neutral detergent fiber**							
Forage type							
PRG	0.96	1.03	1.05	1.08	1.05	1.10	1.04 b
RCPM	0.82	0.87	0.89	0.82	0.90	0.84	0.85 ab
SAM	0.73	0.84	0.77	0.82	0.73	0.78	0.78 a
Mean	0.84	0.91	0.90	0.90	0.89	0.91	
Effects:	Forage type:		*p* < 0.05	SED:			0.078
	Forage allowance:		*p* > 0.05	SED:			0.045
	Interaction:		*p* > 0.05	SED (Within forage type):			0.078
				SED (Between forage types):			0.106
**Acid detergent fiber**							
Forage type							
PRG	0.91 Bab	0.98 ab	0.84 a	0.95 ab	0.97 Bab	1.02 Bb	0.94 b
RCPM	0.87 Bb	0.91b	0.89 b	0.82 b	0.81 Ab	0.64 Aa	0.82 a
SAM	0.72 A	0.81	0.84	0.81	0.80 A	0.78 A	0.79 a
Mean	0.83	0.90	0.86	0.86	0.86	0.81	
Effects:	Forage type:		*p* < 0.01	SED:			0.021
	Forage allowance:		*p* > 0.05	SED:			0.044
	Interaction:		*p* < 0.05	SED (Within forage type):			0.077
				SED (Between forage types):			0.073

## Data Availability

The data presented in this study is available upon reasonable request from the corresponding author.

## Supplementary Materials

The following are available online at https://www.mdpi.com/article/10.3390/ani13081406/s1, Figure S1: Photographs of evaluated forage types, Table S1: Linear regression equations used for estimating forage biomass, Table S2: Nutritive value parameters for forage offered to cows.
