# Supplementary material for: A Forage Allowance by Forage Type Interaction Impacts the Daily Milk Yield of Early Lactation Dairy Cows
Source: Animals (Basel). 2023 Apr 19;13(8):1406. doi: 10.3390/ani13081406 (PMC10135110; doi:10.3390/ani13081406)
Supplement: Supplementary file 1 [file animals-13-01406-s001.zip › animals-2220642-supplementary.pdf]

Supplementary Materials

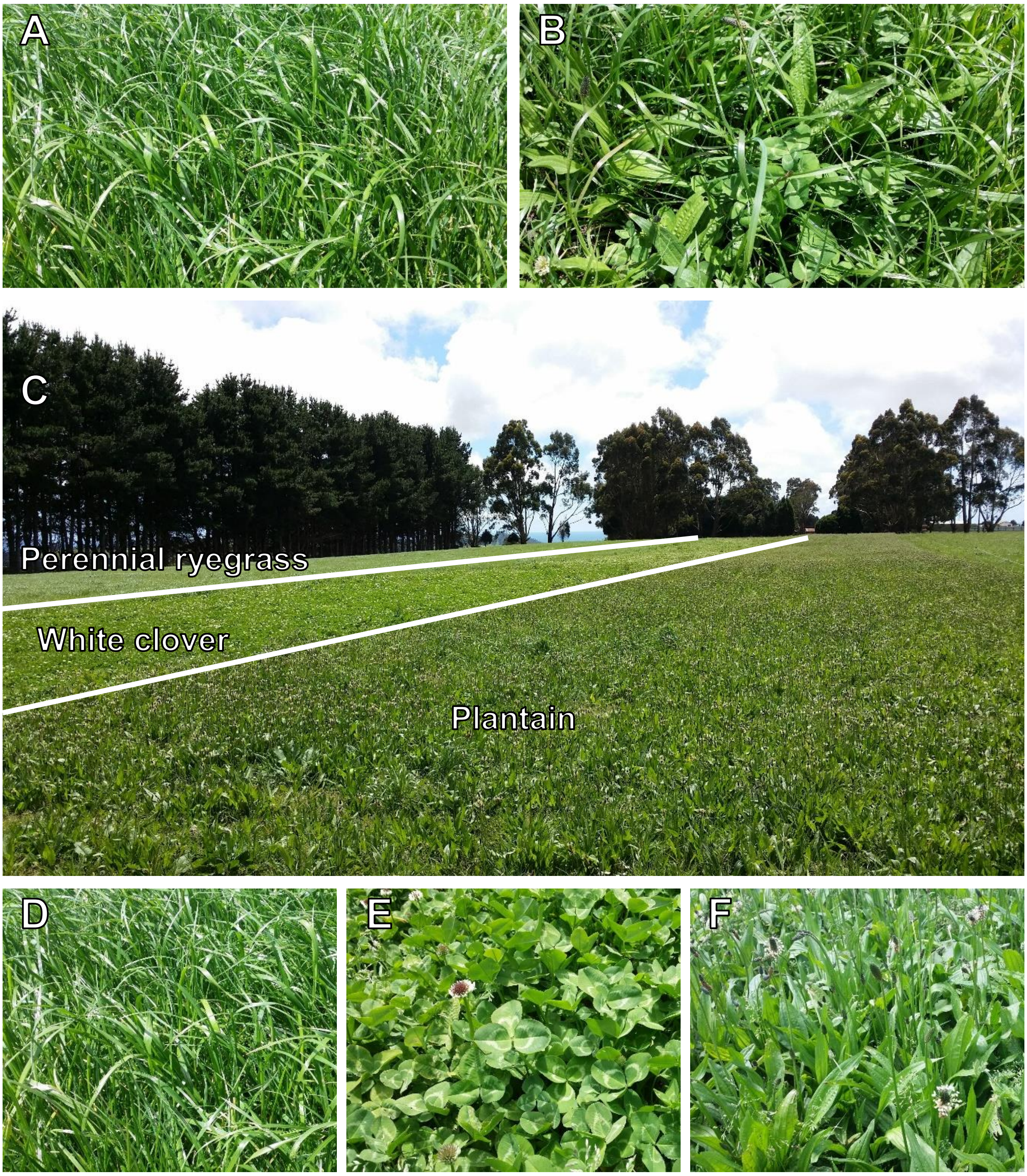

Figure S1. Photographs of evaluated forage types, which included: (A) perennial ryegrass monocultures (PRG); (B) perennial ryegrass, white clover, and plantain mixtures (RCPM); and (C) spatially adjacent monocultures (SAM) of perennial ryegrass (D), white clover (E) and plantain (F).

Table S1 Linear regression equations used for estimating forage biomass (y, kg DM/ha) from compressed forage height (x, mm) measured with an electronic rising plate meter. Abbreviations: PRG, perennial ryegrass monoculture; RCPM, perennial ryegrass, white clover and plantain mixture; SAM, spatially adjacent monocultures of perennial ryegrass, white clover and plantain. Each calibration was developed from 80 individual measurements.

Forage type	Plate meter calibration equation	R^2
PRG and perennial ryegrass component of SAM	$y = 10.8x+393$	68.1
White clover component of SAM	$y = 11.3x+419$	71.3
Plantain component of SAM	$y = 10.0x+390$	82.6
RCPM	$y = 10.6x+390$	59.2

Table S2. Nutritive value parameters for forage offered to cows grazing each forage type, including PRG, perennial ryegrass monoculture; RCPM, perennial ryegrass, white clover and plantain mixture; and SAM, spatially adjacent monocultures of perennial ryegrass, white clover and plantain. Nutritive value of concentrate is also provided. Values are means ± SE (n = 10). Abbreviations include NDFD, neutral detergent fibre digestibility and NDF, neural detergent fibre.

Forage type	PRG	RCPM	SAM			Concentrate
			Perennial Ryegrass	White Clover	Plantain	
Starch (%)	0.5 ± 0.2	0.6 ± 0.2	0.5 ± 0.2	1.4 ± 0.2	1.0 ± 0.2	54.7
Simple sugars (%)	9.0 ± 0.4	11.1 ± 0.5	8.9 ± 0.4	9.0 ± 0.4	12.7 ± 0.4	1.8
Calcium (%)	0.39 ± 0.08	0.72 ± 0.13	0.38 ± 0.06	0.8 ± 0.15	1.07 ± 0.16	1.97
Phosphorus (%)	0.35 ± 0.06	0.35 ± 0.06	0.35 ± 0.06	0.35 ± 0.07	0.35 ± 0.05	0.34
Magnesium (%)	0.18 ± 0.05	0.21 ± 0.05	0.19 ± 0.05	0.25 ± 0.05	0.24 ± 0.06	0.61
Potassium (%)	2.95 ± 0.22	2.72 ± 0.21	3.06 ± 0.26	2.67 ± 0.16	2.62 ± 0.2	0.56
Sodium (%)	0.50 ± 0.13	0.64 ± 0.12	0.48 ± 0.13	0.46 ± 0.14	0.6 ± 0.12	0.45
Iron (mg/kg)	398 ± 5	341 ± 5	388 ± 5	1057 ± 9	486 ± 6	259
Zinc (mg/kg)	20 ± 1	26 ± 1	22 ± 1	31 ± 1	31 ± 1	174
Manganese (mg/kg)	149 ± 2	113 ± 1	131 ± 2	103 ± 2	87 ± 2	134
Sulphur (%)	0.31 ± 0.06	0.38 ± 0.06	0.31 ± 0.06	0.33 ± 0.07	0.41 ± 0.08	0.16
Chloride (%)	1.42 ± 0.14	1.67 ± 0.19	1.62 ± 0.19	1.13 ± 0.18	1.86 ± 0.14	0.78
NDFD (% of NDF)	74.6 ± 0.7	69.2 ± 0.8	73.5 ± 0.7	67.4 ± 0.8	54.5 ± 0.7	47.0
